# Mitochondrial regulation of tumor-associated macrophage function

**DOI:** 10.3389/fimmu.2026.1971517

**Published:** 2026-10-07

**Authors:** Jiabao Xu, Yangtao Liu, Hongjie Zhang, Zhao Yuan, Sheng Yu, Xiaodong Wang, Hanbin Liu, Fei Zhong, Hui Zhang, Han Xiong, Xiaoling Chen, Qingqing Zhang, Jiani Liu, Li Cai, Qizhen Huang, Heying Yue, Liang Huang, Ning Zhu, Yi Yuan, Meng Li, Chengkai Wu

**Affiliations:** 1 Department of Traditional Chinese Internal Medicine, School of Traditional Chinese Medicine, Southern Medical University, Guangzhou, Guangdong, China; 2 Department of Rheumatology and Immunology, Nanfang Hospital, Southern Medical University, Guangzhou, Guangdong, China; 3 The First School of Clinical Medicine, Southern Medical University, Guangzhou, Guangdong, China; 4 The Second Affiliated Hospital of Guilin Medical University, Guilin, China; 5 School of Basic Medical Sciences, Guangzhou University of Chinese Medicine, Guangzhou, Guangdong, China; 6 Department of Nephrology and Rheumatology, The Affiliated Traditional Chinese Medicine Hospital, Guangzhou Medical University, Guangzhou, China; 7 Department of Radiology, the Second Affiliated Hospital, University of South China, Hengyang, Hunan, China; 8 Department of Pharmacy, The Seventh Affiliated Hospital, Southern Medical University, Foshan, Guangdong, China; 9 Department of Rheumatology and Clinical Immunology, The First Affiliated Hospital of Guangzhou Medical University, Guangzhou, China; 10 Institute for Clinical Medical Research, The First Affiliated Hospital of Xiamen University, School of Medicine, Xiamen University, Xiamen, China

**Keywords:** cancer immunotherapy, immunometabolism, lipid droplets, mitochondria, mitochondrial quality control, organelle crosstalk, tumor immune microenvironment, tumor-associated macrophages

## Abstract

Tumor-associated macrophages (TAMs) adapt to hypoxia, nutrient competition, lipid accumulation, and anticancer treatment. Mitochondria contribute to this adaptation through energy metabolism, redox signaling, mitochondrial DNA (mtDNA) sensing, and metabolite-dependent regulation of gene expression. This Review examines mitochondrial dynamics, mitophagy, proteostasis, respiratory remodeling, and lipid-droplet interactions, distinguishing direct TAM evidence from findings in other experimental systems. Tumor studies link these processes to IL-12 and IL-10 production, interferon responses, antigen presentation, and lymphocyte recruitment, with effects that vary by pathway and macrophage state. These findings do not establish a single optimal level of mitochondrial quality control. We discuss how macrophage lineage, spatial niche, and treatment exposure may influence the response to mitochondrial interventions. Therapeutic development will require causal validation in defined TAM populations, selective delivery, and measurements of immune function alongside mitochondrial changes. Evidence in human TAMs and direct studies of organelle contacts remain limited. Somatic mtDNA variation is considered separately from altered gene expression and DNA release, with lineage-tracing evidence distinguished from functional causation.

## 1 Introduction

Tumor-associated macrophages (TAMs) can suppress cytotoxic lymphocytes, promote angiogenesis and matrix remodeling, and contribute to metastasis and resistance to treatments such as immune checkpoint blockade (ICB) (1–4). The classical M1/M2 framework incompletely describes this diversity. Single-cell studies identify overlapping interferon-responsive, antigen-presenting, lipid-associated, complement-rich, angiogenic, hypoxia-adapted, SPP1-positive, and TREM2-positive programs that vary across tumors and patients (5–14). Spatial studies further show that macrophage function depends on cellular origin and location, including proximity to vessels, invasive margins, and lymphocyte-rich regions (15–22).

Mitochondria connect environmental conditions to macrophage function through energy conversion, redox signaling, metabolites, calcium handling, and innate immune sensing. Their maintenance involves fission and fusion, cristae organization, proteostasis, mitochondrial-derived vesicles (MDVs), mitophagy, lysosomal degradation, and biogenesis (23, 24). These processes can influence mitochondrial reactive oxygen species (mtROS), mtDNA release, and inflammatory signaling. Some proteins involved in mitochondrial maintenance also regulate the trafficking or degradation of immune proteins, including major histocompatibility complex (MHC) molecules.

Tumor studies provide specific examples. Myeloid FAM73b/MIGA2 loss promotes fission, alters CHIP-IRF1 regulation, and increases interleukin-12 (IL-12) and antitumor T-cell activity (25). AMPK-dependent Parkin activation promotes ATG5-dependent loss of macrophage MHC-I (26). NDUFA4 repression increases mtDNA-STING signaling and interferon-responsive TAMs (27), whereas complex III-derived oxidants support IL-10 production in the macrophage experiments of Zotta et al. (28). A separate non-tumor macrophage study supports the complex III–IL-10 relationship (29). Reduced activity of the mitochondrial chaperone TRAP1 has also been linked to oxidative metabolism and macrophage-mediated immune suppression (30). These findings indicate that mitochondrial changes have different immune consequences depending on the mechanism and experimental context.

Lipid metabolism and organelle interactions also contribute to mitochondrial regulation in TAMs. Tumor macrophages can accumulate lipid droplets (LDs), upregulate CD36, increase fatty-acid utilization, and use LDs as both buffers against lipotoxicity and reservoirs that sustain oxidative metabolism (31–36). Tumor-derived lipids can move through CD36-dependent routes from extracellular vesicles or lipoproteins into macrophage LDs and then toward mitochondria, supporting metastasis-associated programs (37). In human hepatocellular carcinoma (HCC), LD-laden macrophages show a suppressive phenotype, prolonged survival, and CCL20-dependent recruitment of CCR6-positive regulatory T cells (38). Oxidized LDL can also drive TREM2-positive TAM states through a tumor-macrophage metabolic circuit (39). Although direct tumor evidence for physical LD-mitochondria contact sites remains limited, macrophage studies demonstrate that mitochondria, endoplasmic reticulum, peroxisomes, and LDs assemble into functional multi-organelle units that control lipid flux and inflammatory mediator synthesis (40).

We organize these findings around the interaction between mitochondrial maintenance and immune signaling (**Figure 1**). Clearance of damaged mitochondria may preserve cell function while reducing mtROS or mtDNA exposure; proteins involved in mitochondrial maintenance may also alter immune-cargo trafficking. These effects cannot be ordered on a single scale from insufficient to excessive quality control. Their net consequences depend on the pathway, macrophage population, and immune function assessed. This framework is intended to guide interpretation and has not been validated as a predictive model.

**Figure 1:**
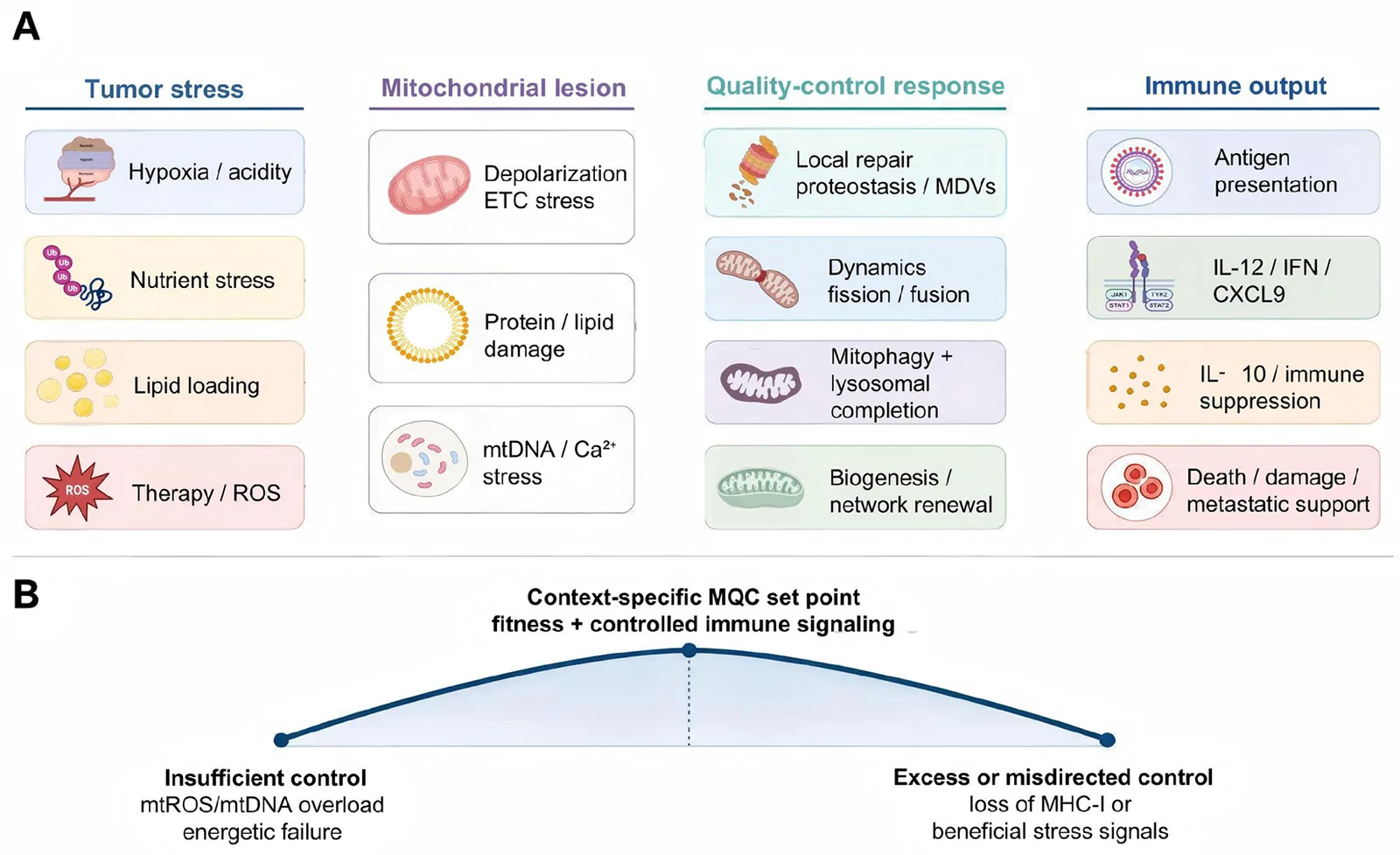
Proposed relationships between mitochondrial state and TAM function. **(A)** Tumor-associated stresses and metabolic inputs can alter mitochondrial signaling, lipid handling, and macrophage immune activity. The illustrated programs overlap and are not mutually exclusive macrophage populations. **(B)** The mitochondrial immune set point is a conceptual model, not a measured optimum or a validated dose-response relationship. The proposed balance between organelle maintenance and immune signaling may vary with macrophage lineage, spatial niche, lipid availability, lysosomal function, and treatment. Individual pathways require independent functional testing.

This Review integrates direct TAM studies, experiments in non-tumor macrophages, and foundational mitochondrial cell biology. We examine how mitochondrial and organelle interactions influence macrophage functions, antigen presentation, and lymphocyte localization, and consider the evidence needed to translate pathway-specific interventions into cancer therapy.

## 2 Mitochondrial states define tumor-associated macrophage functional remodeling

### 2.1 TAM heterogeneity beyond M1/M2 polarization

Single-cell studies identify overlapping inflammatory, interferon-responsive, antigen-presenting, lipid-associated, TREM2-positive, SPP1-positive, hypoxic, angiogenic, and scavenging programs in TAMs (5–7, 16). Several programs can coexist within a tumor or an individual cell population. Embryo-derived tissue-resident macrophages and monocyte-derived macrophages may also occupy different niches and respond differently to treatment (41, 42). This diversity complicates both broad macrophage depletion and extrapolation of a pathway’s effect from one TAM population to another.

Macrophage location affects interactions with lymphocytes. CXCL9- and CXCL10-producing macrophages can support lymphocyte recruitment after ICB (43). In lung cancer models, resident interstitial macrophages near bronchovascular regions supported lymphocyte recruitment and tertiary lymphoid structures, whereas tumor-localized CCL2-associated programs recruited protumor monocyte-derived macrophages (15). In non-small-cell lung cancer (NSCLC), SLC2A1-positive TAM-enriched regions had fewer CD8-positive T cells; TAM-specific Slc2a1 deletion improved CD8 T-cell distribution and anti-PD-L1 efficacy in experimental models (44). These findings support measuring cell location and interactions alongside macrophage markers (45–47).

Systemic metabolism can also alter macrophage function. In obesity models, macrophage PD-1 expression was associated with reduced glycolysis, phagocytosis, and T-cell-stimulatory activity; PD-1 blockade restored aspects of these functions (48). Such findings indicate that nutrient availability and checkpoint signaling should be considered when interpreting metabolic differences between TAM populations.

### 2.2 Spatial and treatment-defined mitochondrial niches

Hypoxic cores, necrotic regions, invasive margins, and perivascular niches expose TAMs to different oxygen and nutrient conditions (49). Hypoxia limits respiratory-chain activity and can induce receptor-mediated mitophagy through BNIP3, NIX, or FUNDC1. Acidity and lactate can also alter substrate use, lysosomal function, and inflammatory signaling (50–54). These conditions may change the response to a mitochondrial intervention. However, direct comparisons of the same perturbation across defined TAM niches are needed to establish when spatial differences produce distinct immune outcomes.

Tumor and immune cells compete for glucose, amino acids, lipids, and oxygen, while macrophages can adapt their substrate use to nutrient availability (55, 56). TAM glucose metabolism has been linked experimentally to spatial T-cell exclusion (44). Regions enriched in oxidized lipoproteins, dying cells, or extracellular vesicles may also favor CD36- or TREM2-associated macrophage programs (37, 39). These observations connect local nutrient conditions to TAM metabolism, although the contribution of individual mitochondrial pathways requires direct testing.

Treatment can alter mitochondrial stress in both tumor and immune cells, release ATP or mtDNA, and change oxygenation and lipid availability (57). The consequences may differ between acute exposure and prolonged treatment (58–61). In HCC models, sorafenib-induced tumor-cell mitochondrial damage increased extracellular mtDNA; macrophage uptake activated TLR9-NF-kappaB signaling and promoted M2-like polarization and drug resistance (62). This example emphasizes the need to identify the cellular source of mitochondrial material when studying treatment-associated TAM responses.

### 2.3 Context-dependent relationships between mitochondrial maintenance and immune function

Four related features can help organize mitochondrial measurements in TAMs: damage burden, maintenance capacity, immune signaling, and immune-cargo handling. Damage includes depolarization, respiratory dysfunction, mtDNA lesions, protein misfolding, and lipid peroxidation (63–66). Maintenance involves dynamics, proteases, antioxidant systems, autophagy, lysosomes, and biogenesis. mtROS, mtDNA, and metabolites can influence NF-kappaB, AP-1, inflammasomes, interferon signaling, or epigenetic enzymes (67–69). Cargo handling includes phagosomal maturation, MHC trafficking, and autophagic degradation. These features need not change in parallel: Parkin can regulate MHC-I loss independently of canonical PINK1-dependent mitophagy (26).

Timing also affects interpretation. Fission may separate damaged mitochondrial regions, whereas persistent fragmentation accompanied by defective clearance may indicate unresolved dysfunction (70–72). Increased mitochondria-lysosome colocalization does not distinguish delivery from completed degradation, and oxygen consumption depends on substrate availability and mitochondrial abundance. Measurements of structure or protein abundance should therefore be paired, where feasible, with turnover, respiratory function, and a defined macrophage immune endpoint. No single measurement currently establishes an optimal mitochondrial state for TAMs.

### 2.4 Experimental settings and definitions

We distinguish findings obtained directly in TAMs from studies of non-tumor macrophages and other cell types. Mitochondrial quality control (MQC) refers here to dynamics, cristae maintenance, proteostasis, MDVs, mitophagy, lysosomal degradation, and biogenesis. Mitophagy specifically denotes selective autophagic delivery of mitochondria to lysosomes. Increased PINK1, Parkin, LC3-II, or mitochondria-lysosome colocalization alone does not establish increased mitophagic flux; fragmentation likewise does not by itself establish mitochondrial damage. These distinctions are relevant when MQC-associated proteins also regulate immune functions independently of mitochondrial turnover.

**Tables 1** and **2** separate experimental models, *in vivo* tumor validation, and human observations. A positive validation entry refers to the specific cited experiment and does not imply that every component in a pathway has been tested in TAMs.

**Table 1:** Representative mitochondrial and organelle-crosstalk mechanisms and their experimental settings.

Module	Representative perturbation and finding	Experimental setting	*In vivo* tumor validation	References
Fission and fusion	Fam73b/Miga2 loss increased fission, stabilized IRF1 through the Parkin–CHIP pathway, and increased IL-12.	Murine macrophages; B16 melanoma and MCA fibrosarcoma	Yes	(25)
Canonical PINK1–Parkin mitophagy	Mitochondrial depolarization recruited Parkin; PINK1-dependent ubiquitin phosphorylation and autophagy-receptor recruitment enabled mitochondrial clearance.	Cultured mammalian cells; foundational organelle studies	No	(88, 89, 92)
Noncanonical Parkin cargo routing	Parkin loss restored macrophage MHC-I; the AMPK–Parkin–ATG5 mechanism was PINK1-independent.	Murine macrophages and transplantable tumor models	Yes	(26)
Receptor-mediated mitophagy	FUNDC1 depletion impaired LC3-associated mitochondrial removal during hypoxia.	Cultured mammalian cells under hypoxia	No	(98)
Mitochondrial integrated stress response	OMA1-dependent DELE1 processing activated HRI and downstream eIF2α–ATF4/CHOP signaling during mitochondrial stress.	Human cell lines and additional non-tumor cell systems	No	(115, 116)
TRAP1 chaperone activity	Myeloid Trap1 loss increased oxidative metabolism and suppressive macrophage activity; TIM4–AMPK signaling reduced TRAP1.	Murine melanoma and lung tumor models	Yes	(30)
Mitochondria-derived vesicles	Macrophage infection induced vesicular delivery of mitochondrial antimicrobial ROS to *Staphylococcus aureus*-containing phagosomes.	Infected macrophages and mouse bacterial-infection experiments	No	(112)
Complex III redox signaling in tumors	S3QEL1.2 reduced IL-10-associated signaling and B16F10 growth; tumor treatment was systemic, with or without local CpG.	LPS-stimulated macrophages; B16F10 melanoma	Yes; systemic drug	(28)
Complex III superoxide in non-tumor macrophages	Uqcrq deficiency reduced TLR-induced IL-10; restoring respiration with alternative oxidase did not rescue this response.	Murine macrophages; influenza and endotoxemia models	No	(29)
Complex IV remodeling	Ndufa4 reduction increased cytosolic mtDNA, STING signaling, and CXCL9-positive interferon-responsive TAMs.	Murine B16F10 melanoma and MC38 colorectal tumors	Yes	(27)
Biogenesis-associated oxidative programming	PGC-1β expression promoted oxidative metabolism and alternative activation while reducing inflammatory cytokine production.	Non-tumor macrophage activation experiments	No	(126)
Lipid uptake and mitochondrial substrate supply	Macrophage Cd36 deletion reduced tumor-derived lipid uptake, oxidative metabolic support, and liver metastasis.	Murine liver-metastasis models and tumor–macrophage cultures	Yes	(37)
Lipid-droplet–organelle contacts	LPS/IFNγ stimulation reorganized organelle contacts; multi-organelle units controlled fatty-acid routing and lipid-mediator production.	Non-tumor macrophages; organelle imaging and lipid-flux experiments	No	(40)

*In vivo* tumor validation is classified for the specific finding and studies cited in each row. Yes indicates a tumor or metastasis experiment in an intact animal; it does not by itself establish macrophage specificity or clinical efficacy. No denotes the absence of an *in vivo* tumor experiment in those cited studies, not absence of evidence throughout the field. Not assessed in the cited study can be used when the listed endpoint was not evaluated.

Genetic targeting with LysM/Lyz2 drivers is myeloid enriched rather than exclusive to TAMs. Where relevant, macrophage depletion, transfer, or alternative lineage controls strengthen attribution. Canonical mitophagy and Parkin-dependent immune-cargo routing are shown separately because their functional readouts differ.

ATF4, activating transcription factor 4; CHOP, C/EBP homologous protein; CpG, CpG oligonucleotide; HRI, heme-regulated inhibitor kinase; IL, interleukin; LC3, microtubule-associated protein 1 light chain 3; MHC, major histocompatibility complex; mtDNA, mitochondrial DNA; ROS, reactive oxygen species; STING, stimulator of interferon genes; TAM, tumor-associated macrophage; TLR, Toll-like receptor.

**Table 2:** Experimental models and human observations in representative macrophage studies.

Study	Cancer model or tissue	Perturbation and finding	*In vivo* tumor validation	Human tumor observations
Gao et al. (25)	B16 melanoma; MCA fibrosarcoma	Fam73b/Miga2 deletion increased IL-12 and T-cell-mediated tumor control through the Parkin–CHIP–IRF1 pathway.	Yes; genetic models	No
Wang et al. (26)	MC38 colorectal, Hepa1–6 HCC, CT2A glioma, and E0771 breast tumors	Parkin loss preserved macrophage MHC-I, improved antitumor immunity, and enhanced checkpoint blockade.	Yes; systemic and macrophage-focused perturbations	Yes; public tumor and immunotherapy transcriptomic associations
Clark et al. (27)	B16F10 melanoma and MC38 colorectal tumors; additional systemic-knockout models	Ndufa4 reduction promoted mtDNA–STING signaling and interferon-responsive TAMs; miR-147 delivery enhanced anti-PD-1 activity.	Yes; myeloid genetics and local RNA delivery	Yes; pan-cancer single-cell expression of C15orf48 in interferon-responsive TAMs
Zotta et al. (28)	B16F10 melanoma; LPS-stimulated macrophages	Complex III redox suppression lowered IL-10-associated signaling; systemic S3QEL1.2 reduced tumor growth, including with intratumoral CpG.	Yes; systemic S3QEL1.2	No; human blood-derived macrophages were studied, not human tumors
Stoolman et al. (29)	Non-tumor murine macrophages; influenza and LPS endotoxemia	Uqcrq loss reduced IL-10; alternative oxidase restored respiration but not IL-10, whereas PKA activation rescued IL-10 release.	No	No
Zhao et al. (30)	YUMM1.7/B16F10 melanoma and LLC lung tumors; human colon cancer	Myeloid Trap1 loss increased suppressive oxidative metabolism; tumor-associated TIM4–AMPK signaling reduced TRAP1.	Yes; myeloid Trap1 perturbation	Yes; TRAP1 and macrophage-marker staining in colon tumors
Wang et al. (38)	Human HCC; Hepa1–6 subcutaneous and orthotopic tumors	Combined DGAT1/2 inhibition reduced lipid-droplet accumulation, CCL20-associated Treg recruitment, and tumor growth.	Yes; combined DGAT inhibition	Yes; HCC macrophage phenotyping and tissue observations
Yang et al. (37)	LLC liver metastasis; additional B16F10, Hepa1-6, and CT26 metastasis models	Macrophage Cd36 deletion reduced tumor-lipid uptake and liver metastasis and restored CD8 T-cell activity.	Yes; macrophage Cd36 deletion	Yes; human liver-metastasis transcriptomic associations
Chu et al. (39)	Spontaneous HCC and orthotopic Hepa1–6 tumors; human HCC	Tumor SQLE-dependent oxLDL supported TREM2–SYK–CEBPα signaling and suppressive TAMs; pathway interruption improved tumor immune responses.	Yes; tumor and macrophage-pathway perturbations	Yes; single-cell/spatial, prognosis, and immunotherapy-response associations
Yang et al. (62)	Hepa1–6 orthotopic, SNU739 xenograft, and DEN-induced HCC	Tumor-derived mtDNA activated macrophage TLR9–NF-κB; TLR9-pathway interruption reduced suppressive polarization and improved sorafenib activity.	Yes; genetic and pharmacologic pathway tests	Yes; HCC TLR9/CD163 tissue observations and sorafenib-response datasets
Wang et al. (44)	LLC and TC-1 tumors; human NSCLC	Myeloid Slc2a1 loss and SLC2A1 inhibition improved CD8 T-cell function and spatial distribution and enhanced anti-PD-L1 responses.	Yes; myeloid deletion, TAM transfer, and inhibitor studies	Yes; NSCLC spatial observations and immunotherapy-response associations
Mansouri et al. (189)	Multiple mouse lung tumor models; human lung tumors and precision-cut tumor slices	IRG1/itaconate restrained tumor growth through G6PD/PPP inhibition and effects on both macrophages and tumor cells.	Yes; Irg1 genetics, bone-marrow chimeras, and 4-octyl itaconate	Yes; tumor profiling and ex vivo precision-cut tumor slices
Zhang et al. (190)	Hepa1–6 subcutaneous/orthotopic and hydrodynamic spontaneous HCC	Myeloid Bcat1 loss increased crotonate/H3K14 crotonylation and suppressive lipid programs; BCAT1 restoration or macrophage transfer improved antitumor responses.	Yes; lineage controls, genetic perturbation, and macrophage transfer	Yes; HCC single-cell data, isolated macrophages, and multiplex tissue staining

Yes/No refers only to the cited study. *In vivo* tumor validation requires an intact-animal tumor or metastasis experiment; it does not establish TAM-specific causation or clinical efficacy. Infection models, cell culture, and ex vivo slices do not meet this definition.

Human observations include patient tissue, tumor-derived cells or slices, and patient datasets; human cell lines and healthy-donor macrophages alone are excluded. Associations and ex vivo perturbations are identified separately.

Zotta et al. (28) tested systemic S3QEL1.2 with or without CpG in B16F10 tumors; this was not TAM-selective treatment. Stoolman et al. (29) studied non-tumor inflammatory models.

DEN, diethylnitrosamine; DGAT, diacylglycerol acyltransferase; G6PD, glucose-6-phosphate dehydrogenase; HCC, hepatocellular carcinoma; LLC, Lewis lung carcinoma; NSCLC, non-small-cell lung cancer; oxLDL, oxidized LDL; PPP, pentose phosphate pathway; Treg, regulatory T cell.

## 3 Mitochondrial quality control and genome maintenance

### 3.1 Fission, fusion, and cristae remodeling

Mitochondrial fission and fusion form the structural layer of MQC. DRP1 is recruited from the cytosol to the outer mitochondrial membrane by receptors including MFF, where it oligomerizes and constricts the organelle (73–78). Fusion requires mitofusins on the outer membrane and OPA1 on the inner membrane (79). OPA1 additionally maintains cristae architecture and cytochrome c sequestration independently of gross network fusion (80). These structural transitions alter the topology of electron transport, substrate exchange, mitochondrial positioning, and the segregation of damaged segments. In macrophages, TLR4 stimulation can induce DRP1-dependent fission as an instructed immune response rather than as a passive marker of injury (81).

A tumor-specific example is the MIGA2/FAM73b pathway. Myeloid Fam73b loss shifted mitochondria toward fission, altered Parkin-dependent protein turnover, stabilized IRF1 through the CHIP axis, increased IL-12, and strengthened T-cell-mediated tumor control (25). The accompanying changes in IRF1 and IL-12 link morphology to immune function in this model. They do not establish that mitochondrial fragmentation generally favors antitumor activity.

Cristae structure deserves separate attention. Changes in OPA1 processing, MICOS organization, inner-membrane lipid composition, and respiratory supercomplex assembly can alter electron transport chain (ETC) efficiency, ROS topology, cytochrome c mobilization, and mtDNA containment without large changes in total mitochondrial area (82–86). Measurements of cristae organization and respiratory function can therefore complement whole-organelle morphology when testing mechanisms involving ETC remodeling or mtDNA release (**Figure 2**).

**Figure 2:**
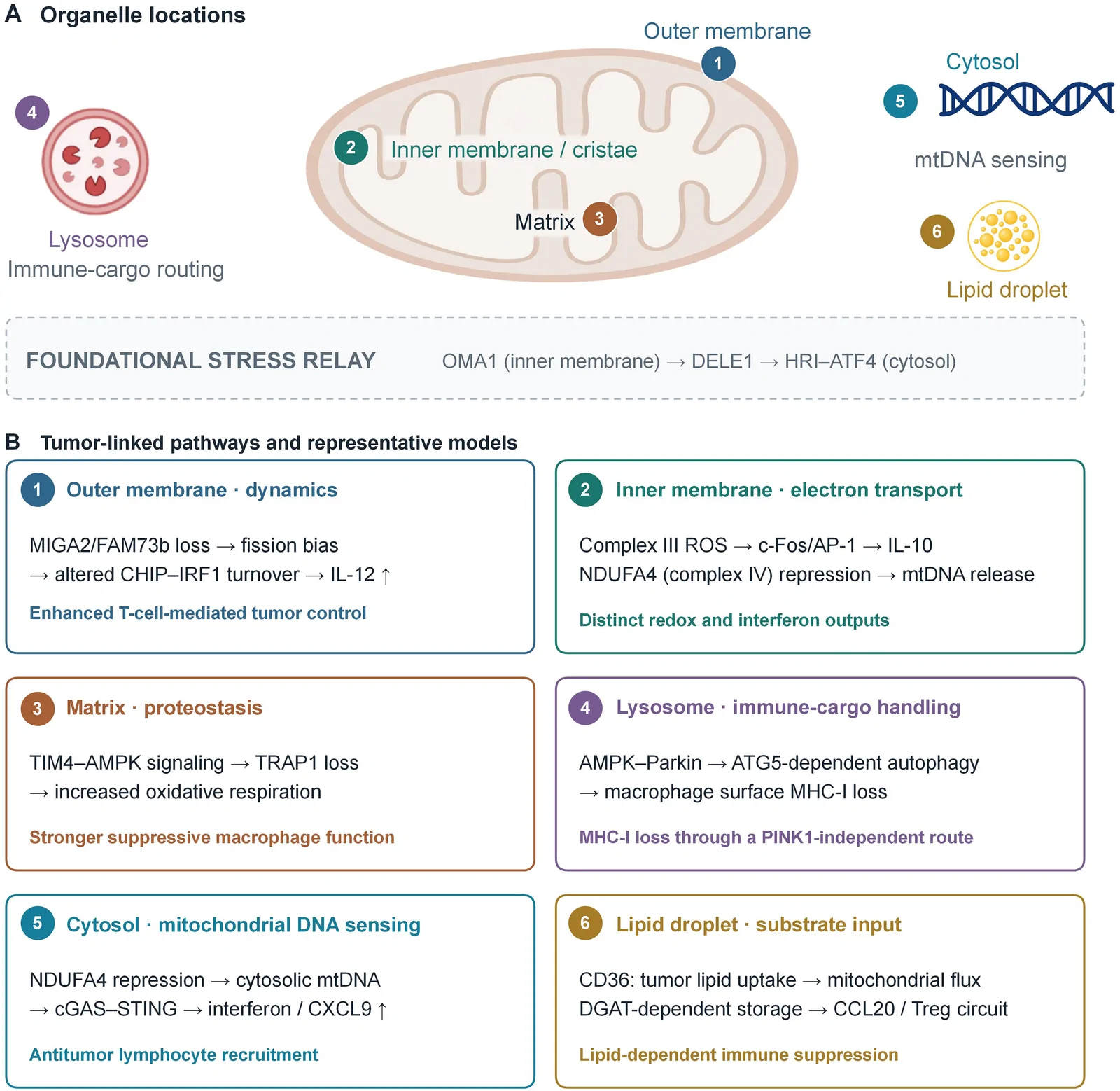
Compartment-specific mitochondrial regulation in tumor-associated macrophages. **(A)** A localization map distinguishes the mitochondrial outer membrane, inner membrane/cristae, matrix, lysosome, cytosol, and lipid-droplet compartment. Numbered markers correspond to the pathway cards in panel **(B)** Dashed boxes identify foundational cell-biological mechanisms whose tumor-specific immune consequences require additional testing. The OMA1-DELE1-HRI-ATF4 pathway relays mitochondrial stress to the cytosolic integrated stress response (115, 116). **(B)** Representative tumor-linked mechanisms and cancer models are assigned to the compartment in which the principal regulatory event occurs (25–30, 37, 38). Compartment placement does not imply that the complete pathway is confined to that organelle or that the same effect occurs across cancers. The Parkin-MHC-I pathway concerns noncanonical immune-cargo routing and is PINK1 independent (26). Complex III redox signaling is supported by activated-macrophage experiments, with systemic S3QEL1.2 treatment tested in B16F10 melanoma (28, 29). CD36-dependent lipid uptake and DGAT-dependent storage are distinct upstream modules; demonstrated lipid flux does not establish a requirement for physical lipid-droplet-mitochondria contact sites. CRC, colorectal cancer; HCC, hepatocellular carcinoma; MCA, methylcholanthrene; MQC, mitochondrial quality control.

### 3.2 Mitophagy and noncanonical immune-cargo routing

In canonical PINK1-Parkin mitophagy, loss of membrane potential prevents PINK1 import and degradation, allowing PINK1 to accumulate on the outer mitochondrial membrane (87). PINK1 phosphorylates ubiquitin and Parkin, creating a feed-forward signal that recruits autophagy receptors and LC3-positive membranes (88–96). PINK1 also facilitates segregation of damaged regions by removing inhibitory PKA signaling and permitting DRP1-dependent fission (97). Alternative receptors, including FUNDC1, NIX/BNIP3L, BNIP3, prohibitin 2, and externalized cardiolipin, can engage LC3-family proteins under hypoxia or membrane stress (98–102).

Insufficient mitochondrial clearance can increase mtROS, oxidized mtDNA, and inflammasome signaling. Autophagy proteins can restrict mtDNA release and NLRP3 activation (103, 104), and suppression of PINK1-dependent mitophagy can intensify macrophage inflammatory function in non-tumor settings (105). These observations provide a mechanistic rationale for transiently preserving mitochondrial danger signals, but they do not imply that chronic mitophagy failure will be beneficial in tumors. Persistent damaged mitochondria can compromise ATP generation, phagocytosis, viability, and antigen processing.

In mouse tumor models, Parkin loss enhanced antitumor responses and improved checkpoint blockade. The reported mechanism involved AMPK-dependent, PINK1-independent Parkin activation and ATG5-dependent loss of macrophage MHC-I (26). This result concerns autophagic handling of immune cargo and does not establish that canonical mitophagy has the same effect. Mitochondrial turnover and MHC-I degradation therefore need to be evaluated separately.

Mitophagy assays measure different steps in mitochondrial turnover. The pH-sensitive reporters mt-Keima and mito-QC detect mitochondrial delivery to acidic compartments (106, 107). Pairing these reporters with lysosomal perturbation and measurements of mitochondrial mass helps distinguish delivery from degradation and net organelle loss. Experimental considerations for interpreting MQC perturbations are summarized in **Table 3**.

**Table 3:** Experimental considerations for interpreting mitochondrial pathways in TAMs.

Claim being tested	Measurements and controls	Interpretive limit
Increased mitophagy	Combine mt-Keima or mito-QC with lysosomal perturbation, mitochondrial mass, and complementary genetic disruption (106, 107).	Acidic-compartment delivery, lysosomal degradation, and net mitochondrial loss are different measurements.
Fission or cristae remodeling	Quantify network morphology and DRP1 recruitment; assess mitochondrial mass. Add cristae imaging and respiratory measurements when the proposed mechanism involves the inner membrane (73–86).	Fragmentation alone does not establish damage or a direction of immune function.
Respiratory or redox mechanism	Measure oxygen consumption, coupling, membrane potential, and mitochondrial mass; use source-selective perturbation when assigning ROS to complex III (28, 29).	A whole-cell ROS signal or reduced respiration cannot localize the relevant source.
Lipid uptake or oxidation	Combine uptake and storage measurements with isotope tracing, genetic controls, and exposure-matched pharmacology (37, 38, 156).	LD abundance is not a measure of fatty-acid oxidation; etomoxir can have CPT1-independent effects.
Macrophage-specific antitumor effect	Pair macrophage functional assays with lineage-restricted tumor perturbation, rescue or complementary depletion/adoptive approaches, and human observations where available (25–27).	Tumor growth alone does not establish a TAM mechanism; myeloid Cre lines may affect other cells.
Causal somatic mtDNA variant	Resolve genotype and heteroplasmy in TAMs and matched blood/tumor cells; control contamination and acquired mitochondrial material; test defined variants on matched nuclear backgrounds (133–137).	Clonal association, oxidative DNA damage, mtDNA release, and an inherited variant are not evidence of a naturally acquired TAM-specific causal mutation.

These are considerations for evaluating causal claims, not a checklist already completed by every cited study. Experimental requirements depend on the claim and model. MQC, mitochondrial quality control; TAM, tumor-associated macrophage; LD, lipid droplet.

### 3.3 Proteostasis, local repair, and mitochondrial stress signaling

Local protein repair and selective cargo removal can occur alongside mitophagy. Matrix and inner-membrane proteases remove damaged proteins, chaperones refold imported polypeptides, and the ubiquitin-proteasome system extracts selected outer-membrane substrates. MDVs can transport oxidized or damaged cargo to lysosomes without eliminating the parent organelle (108–113). PINK1 and Parkin participate in some MDV pathways, illustrating how the same proteins can coordinate local repair and whole-organelle clearance (114).

Mitochondrial stress is also relayed to the cytosol and nucleus. OMA1-dependent cleavage of DELE1 activates HRI-eIF2alpha-ATF4 signaling, providing a direct route from inner-membrane stress to translational control (115–118). In TAMs, this integrated stress response could influence amino-acid metabolism, antioxidant defense, cytokine translation, and survival; however, direct lineage-restricted tumor evidence remains limited. Whether this pathway supports transient adaptation or sustained suppressive activity in TAMs requires direct testing.

TRAP1 provides a recent tumor-specific example of mitochondrial proteostasis intersecting with immune metabolism. In 2025, TRAP1 was identified as a mitochondrial chaperone checkpoint that restrains oxidative phosphorylation (OXPHOS) and suppressive macrophage activity. Tumor-associated TIM4-AMPK signaling reduced TRAP1, increasing ETC activity and favoring an immunoinhibitory state (30). These findings link a mitochondrial chaperone to respiratory and immune changes without establishing that altered organelle turnover is required.

### 3.4 Respiratory-chain remodeling and compensatory biogenesis

ETC composition and the sites of electron leak can influence immune signaling (119–121). Activated-macrophage experiments link complex III-derived ROS to IL-10 production (28, 29). In the study by Zotta et al., systemic S3QEL1.2 treatment also reduced B16F10 tumor growth (28); that tumor response was not shown to be exclusively macrophage-dependent. NDUFA4 repression in TAMs remodeled complex IV, increased cytosolic mtDNA, activated STING, expanded CXCL9-positive interferon-responsive TAMs, and improved ICB responses (27). The immune effects of these interventions depend on the respiratory component, downstream pathway, and cells affected.

Biogenesis replenishes mitochondrial capacity after stress or clearance (122–125). AMPK, SIRT1, PGC-1 coactivators, nuclear respiratory factors 1 and 2, and TFAM coordinate nuclear and mitochondrial programs, and PGC-1beta-associated oxidative metabolism can restrain inflammatory activation in non-tumor macrophages (126). In cancer, however, increased mitochondrial mass can also support the survival of suppressive TAMs. Mitochondrial mass should therefore be interpreted alongside turnover, substrate use, and immune function.

Mitochondrial biogenesis within a macrophage is distinct from the acquisition of mitochondria from another cell. Transfer has been described in several non-tumor settings (127–130). A preclinical delivery study used surface-engineered mitochondria to alter TAM phenotype and improve antitumor responses (131); this is an engineered intervention, not evidence that the same transfer occurs spontaneously in human tumors. Separately, tumor-to-T-cell transfer of mutation-bearing mitochondria impaired T-cell function (132). Because that study examined T cells, it does not establish a corresponding TAM mechanism. The new Section 3.5 discusses mitochondrial genotype, whereas Section 5.2 addresses the sensing of released mtDNA.

### 3.5 Somatic mitochondrial variation and TAM function

Genetic variation provides a potential source of persistent mitochondrial differences between TAMs. Mutations in mtDNA should be distinguished from alterations in nuclear genes encoding mitochondrial proteins. For example, NDUFA4 repression remodels complex IV in TAMs, but this finding does not demonstrate a somatic mtDNA mutation (27). Likewise, increased mtDNA release, oxidative lesions, or altered copy number do not establish a change in mitochondrial sequence. For a heteroplasmic variant, the proportion of mutant genomes is also relevant: experiments introducing truncating Mt-Nd5 variants into melanoma cells showed that complex I disruption and associated metabolic changes depended on mutant abundance (133). These relationships cannot yet be assumed to have the same thresholds or consequences in macrophages.

Evidence that mitochondrial genotype can alter macrophage function comes mainly from non-tumor models. Macrophages from Polg proofreading-deficient mice exhibited mtDNA instability, enhanced interferon and inflammatory responses, and partial normalization after disruption of cGAS-STING-interferon signaling (134). POLG is nuclear encoded, and this systemic mutator model therefore differs from a naturally acquired, macrophage-restricted mtDNA variant. More directly, macrophages carrying the inherited heteroplasmic mitochondrial tRNA-Ala variant m.5019A>G showed impaired oxidative phosphorylation and altered cytokine responses after lipopolysaccharide stimulation (135). This establishes a functional effect of a defined mtDNA mutation in macrophages, but neither a somatic origin nor an effect in the tumor microenvironment. Together, these findings justify testing whether TAM mtDNA variants affect respiratory capacity, inflammatory responsiveness, and antigen presentation, without assigning a uniform antitumor or immunosuppressive direction.

Somatic mtDNA variants have also become useful markers of TAM ancestry. Building on single-cell mitochondrial lineage tracing (136), Liu and colleagues jointly profiled mitochondrial variants and chromatin accessibility in human lung and ovarian cancers. Their Mitotrek analysis connected intratumoral macrophages and type 3 dendritic cells to circulating monocytes and identified clones with different differentiation biases (137). In this setting, mtDNA variants function as endogenous lineage barcodes. A clone’s association with a macrophage state does not show that its barcode mutation caused that state; inherited epigenetic properties, other genetic alterations, and local exposures remain alternative explanations.

Tumor-cell mutations provide a separate route to macrophage remodeling. In melanoma models, engineered cancer-cell Mt-Nd5 mutations changed the immune microenvironment, including the distribution of macrophage states, and improved checkpoint-blockade responses through a neutrophil-dependent mechanism (133). Thus, macrophage changes can arise downstream of a mutation in another cell type.

The studies considered here leave unresolved whether naturally acquired somatic mtDNA variants within TAMs directly determine their immune functions *in vivo*. Addressing this question will require sequencing purified or individually resolved TAMs together with matched blood and tumor cells, quantifying heteroplasmy, and separating stable macrophage genotypes from ambient DNA, doublets, engulfed material, and transferred mitochondria. Functional experiments should compare defined variants across mutant burdens on a matched nuclear background and measure respiration alongside antigen presentation, cytokine production, and lymphocyte responses. Macrophage-restricted perturbation and rescue in tumor models would help distinguish a causal mitochondrial variant from a lineage marker or a consequence of tumor-induced stress.

## 4 Lipid-droplet-mitochondria crosstalk in TAM immunometabolism

### 4.1 Lipid droplets as buffers, reservoirs, and survival organelles

Lipid loading is a defining pressure in many TAM populations (138). LDs can buffer potentially toxic fatty acids, provide substrates for membrane synthesis, and store neutral lipids that can later be mobilized for signaling or oxidation. In human and murine tumors, LD-dependent fatty-acid metabolism has been linked to suppressive macrophage differentiation (31), and CD36-dependent lipid uptake can increase fatty-acid oxidation, OXPHOS, ROS, and STAT6-associated programs (32). These findings place lipid handling upstream of mitochondrial state rather than treating LD accumulation as a passive histological marker (139–142).

A 2024 HCC study reported that LD-laden macrophages were enriched for TREM2, PD-L1, CD206, and CD163, suppressed CD8 T-cell activity, and persisted longer in tumors. LD accumulation promoted CCL20 secretion and recruitment of CCR6-positive regulatory T cells; inhibiting triglyceride synthesis through DGAT1/2 reduced this circuit and delayed tumor growth (38). The reported effects involved macrophage persistence and Treg recruitment, in addition to lipid storage.

The consequences of lipid accumulation also depend on the source of lipid and the cellular response to it. Apoptotic cancer cells can suppress 5-lipoxygenase in TAMs, altering an enzyme involved in lipid-mediator synthesis (143). This is distinct from measuring neutral-lipid storage or mitochondrial fatty-acid oxidation. LD abundance alone does not identify lipid origin, turnover, or immune function; tracer studies can distinguish these possibilities.

### 4.2 Physical organelle contacts and multi-organelle units

Efferocytosis can alter macrophage nutrient transport and metabolism. Studies have linked apoptotic-cell uptake to glucose transport and lactate release, arginine metabolism, and a metastasis-supporting environment during mammary gland involution (144–146). These findings do not by themselves demonstrate physical LD-mitochondria contacts. In non-tumor macrophages, multispectral imaging identified mitochondria-ER-LD and mitochondria-ER-peroxisome-LD units associated with fatty-acid flux and inflammatory lipid metabolism (40). ER-mitochondria contacts also participate in autophagosome formation in other cell systems (147). Whether these contacts causally regulate the corresponding immune functions in TAMs remains to be tested.

MIGA2 is relevant to this question because it participates in mitochondrial fusion-related biology and has also been implicated in organelle contact and lipid metabolism outside the TAM literature. The tumor study linking MIGA2 loss to fission, Parkin-CHIP-IRF1 signaling, and IL-12 provides direct immune evidence (25), but it does not by itself establish that LD-mitochondria contacts mediate the phenotype. Future TAM experiments should combine contact-site imaging with isotope tracing and selective perturbation of candidate tethers to determine whether contact duration or composition predicts lipid oxidation, mtROS, or immune function.

### 4.3 Lipid transfer, OXPHOS, and TREM2-associated suppressive states

Tumor-macrophage lipid transfer can directly feed mitochondrial metabolism. In liver metastasis, tumor extracellular vesicles transferred long-chain fatty acids to CD36-positive metastasis-associated macrophages; lipids moved from LDs toward mitochondria, increased respiration, and supported metastatic progression. Macrophage CD36 loss restored CD8 T-cell immunity and reduced metastasis (37). This provides a mechanistic bridge from extracellular lipid availability to LD storage, mitochondrial substrate flux, and immune suppression.

A 2025 HCC study identified a related oxLDL-TREM2 circuit. A metabolically defined tumor-cell archetype increased oxidized LDL production, and oxLDL promoted TREM2-positive TAM polarization through a TREM2-SYK-CEBPalpha pathway, generating an immune-excluded niche and poorer immunotherapy response (39). Although this mechanism does not prove that mitochondrial oxidation is required at every step, it supports the broader concept that the chemical form of lipid input - free fatty acid, triglyceride, oxidized lipoprotein, or vesicular cargo - can specify different TAM programs.

Additional lipid-laden macrophage populations, including FABP7-associated states, have been linked to premetastatic niche formation and liver metastasis (148). These observations suggest that interventions should specify the substrate and trafficking step they target. CD36 uptake, DGAT-dependent storage, lipolysis, acyl-CoA activation, contact-site transfer, and mitochondrial entry are separable nodes with different consequences for lipotoxicity and immune function. Related myeloid work further shows that lipid-transport proteins can enforce suppressive metabolic programs outside macrophages, reinforcing the need for lineage-specific validation (149).

### 4.4 Lipid peroxidation, iron stress, and ferroptotic reprogramming

Iron-dependent lipid peroxidation contributes to ferroptotic cell death and is regulated by GPX4 and membrane lipid composition (150–153). In tumor models, CD8 T-cell-derived interferon-gamma can promote cancer-cell ferroptosis (154). Separately, ferroptosis-strengthened nanotherapy has been reported to shift TAM metabolism toward glycolysis and increase tumoricidal activity (155). These findings involve different cellular targets. Interventions that alter lipid peroxidation require direct assessment of macrophage function, tumor-cell death, and effects on other immune and normal cells.

### 4.5 Experimental assessment of lipid handling

Pharmacologic interpretation in this field requires caution. Etomoxir, widely used to infer CPT1-dependent fatty-acid oxidation, can disrupt coenzyme A homeostasis at commonly used concentrations and alter macrophage activation independently of mitochondrial fatty-acid entry (156). Strong claims should therefore use orthogonal inhibitors, genetic perturbation, and isotope tracing. Stable-isotope labeled glucose, glutamine, fatty acids, or tumor-derived lipids can determine whether mitochondrial carbon comes from uptake, LD mobilization, or *de novo* synthesis. Imaging should distinguish LD abundance from contact frequency and, when possible, quantify contact duration or proximity together with mitochondrial potential, respiration, and lipid peroxidation in the same cell state.

## 5 Mitochondrial signals regulating TAM function

### 5.1 Redox signaling

mtROS can influence macrophage signaling, with effects that depend on the stimulus and site of production. Complexes I and III generate superoxide that can affect redox-sensitive signaling proteins. In non-tumor macrophage models, TLR signaling increases mitochondrial recruitment to phagosomes and bactericidal ROS (157); mitochondrial ROS has also been implicated in NLRP3 activation and inflammatory cytokine production (158, 159). Zotta et al. linked complex III-derived ROS to c-Fos/AP-1 activity and IL-10 production and also tested systemic redox modulation in a tumor model (28). Stoolman et al. demonstrated the complex III–IL-10 relationship in non-tumor macrophage and inflammatory models (29). These findings do not support a uniformly inflammatory or antitumor effect of mtROS.

Mitochondrial antioxidants may alter both oxidative injury and redox-dependent signaling. Experiments should distinguish mitochondrial from total-cell ROS and, where possible, identify the producing site. Mitochondrial function, cell viability, and the relevant cytokine or lymphocyte response should be assessed together to determine the consequence of ROS modulation in a defined macrophage population.

### 5.2 Mitochondrial DNA sensing

mtDNA can activate innate immune receptors when it reaches the appropriate cellular compartment (63, 160). Cytosolic DNA activates cGAS-STING (161–163), whereas extracellular tumor-derived mtDNA can be internalized and engage TLR9 in HCC-associated macrophages (62). Mitochondrial DNA synthesis and autophagy-dependent control of mtDNA availability also contribute to inflammasome activation (68, 103, 104). Mitochondrial DNA stress can induce interferon responses (164), whereas apoptotic caspases can limit this signaling (165, 166). Inner-membrane permeabilization and BAX/BAK macropores provide mechanisms for mtDNA release during cell stress or apoptosis (167, 168).

TAM studies illustrate different consequences of mtDNA sensing. NDUFA4 repression increased cytosolic mtDNA, activated STING, and enhanced CXCL9-positive interferon-responsive programs (27). In sorafenib-treated HCC models, extracellular tumor-derived mtDNA was taken up by macrophages and activated TLR9-NF-kappaB signaling, promoting M2-like polarization and drug resistance (62). Thus, studies of mtDNA-directed interventions should identify its cellular source, recipient-cell compartment, and sensing receptor; increased mtDNA release alone does not predict an antitumor response.

Mutation status and DNA sensing should be assessed separately. NDUFA4-dependent mtDNA release in TAMs and mtDNA-stress experiments establish that changes in mitochondrial integrity can activate DNA-sensing pathways without identifying a causal mtDNA sequence variant (27, 164). Conversely, the presence of a mitochondrial variant does not establish that DNA has escaped the organelle or reached a receptor-accessible compartment. Studies attributing altered TAM signaling to mtDNA mutations should therefore measure genotype and heteroplasmy together with DNA localization and receptor dependence.

### 5.3 Metabolite signaling

Mitochondrial metabolites influence transcription and chromatin regulation (169–171). In macrophage models, succinate supports HIF-1alpha-dependent IL-1beta production (172), while broader metabolic programs accompany changes in activation state (173). Itaconate inhibits succinate dehydrogenase and can activate NRF2 through KEAP1 modification (174, 175); alpha-ketoglutarate influences dioxygenases and macrophage activation (176–181). Metabolite abundance should be interpreted separately from pathway flux because intracellular pools can reflect synthesis, uptake, consumption, or compartmental exchange (182–184).

Macrophage metabolism also affects tumor clearance and treatment response (185–188). In lung cancer, spatial metabolomics showed lower itaconate in tumor regions, with macrophages constituting the principal IRG1-expressing population. Genetic and pharmacologic experiments indicated that IRG1/itaconate restrained tumor growth and altered macrophage function partly through inhibition of glucose-6-phosphate dehydrogenase and pentose-phosphate-pathway flux (189). This study links the spatial distribution of a metabolite to a tested macrophage mechanism.

Metabolic regulation of chromatin can affect TAMs through pathways outside conventional MQC. In HCC, BCAT1 constrained intracellular crotonate and histone crotonylation. BCAT1 loss increased lipid-metabolism genes and immunosuppressive TAM features, whereas myeloid BCAT1 overexpression improved antitumor immunity and anti-PD-1 responses (190). TRAP1 has also been linked to mitochondrial respiration, metabolite balance, and epigenetic regulation (30). These studies connect metabolic changes to gene regulation without establishing a shared mechanism across all TAM states.

## 6 Macrophage immune functions downstream of mitochondrial regulation

### 6.1 Antigen presentation and T-cell communication

Antigen presentation depends on energy supply, phagosomal maturation, proteolysis, peptide loading, and membrane trafficking. Mitochondrial metabolism may influence these processes, but autophagy-related perturbations are not specific to mitochondrial turnover. LC3-associated phagocytosis (LAP), canonical autophagy, and MHC trafficking share parts of the cellular machinery (191–193). Phagosomal redox regulation also affects antigen processing, as shown in dendritic cells (194). Changes after ATG5, LC3, or lysosomal perturbation therefore require experiments that distinguish these processes from mitophagy.

AMPK-dependent Parkin activation promotes ATG5-dependent macrophage MHC-I loss in tumor models; this effect requires Parkin catalytic activity but is PINK1-independent (26). A separate study identified roles for PINK1 and Parkin in mitochondrial antigen presentation outside the TAM setting (195). These findings should be distinguished mechanistically. Parkin expression alone is therefore insufficient to infer mitophagic activity or antigen-presentation capacity. Studies of Parkin-directed interventions should assess surface MHC-I, antigen-specific T-cell responses, mitochondrial damage, and mitophagic flux in the same experimental setting.

MHC-II expression has been studied directly in TAMs (196), but its dependence on mitochondrial pathways remains less well defined. MHC-II presentation involves CIITA, invariant-chain processing, HLA-DM-mediated peptide exchange, lysosomal pH, and cathepsins. Mitochondrial effects on lysosomal function offer possible links that require TAM-specific testing. Studies relating lipid accumulation to impaired antigen presentation in dendritic cells provide adjacent evidence, rather than proof of a TAM MHC-II mechanism (197, 198). Peptide-specific presentation assays and antigen-specific T-cell responses would help establish whether mitochondrial perturbation changes functional presentation.

### 6.2 Spatial organization of macrophage and lymphocyte responses

Several mitochondrial pathways converge on cytokine production or macrophage-lymphocyte communication, but their reported effects arise in different experimental settings (**Figure 3**). MIGA2 loss increases IRF1-associated IL-12 production (25), while NDUFA4 repression increases mtDNA-STING-CXCL9 signaling (27). Complex III ROS supports IL-10 (28), and Parkin activation promotes macrophage MHC-I loss (26). Reduced TRAP1 activity favors suppressive oxidative metabolism (30). Lipid-loaded and oxLDL-responsive macrophages also support suppressive functions (38, 39). These observations define pathway-specific examples; they do not establish mutually exclusive mitochondrial classes of TAMs.

**Figure 3:**
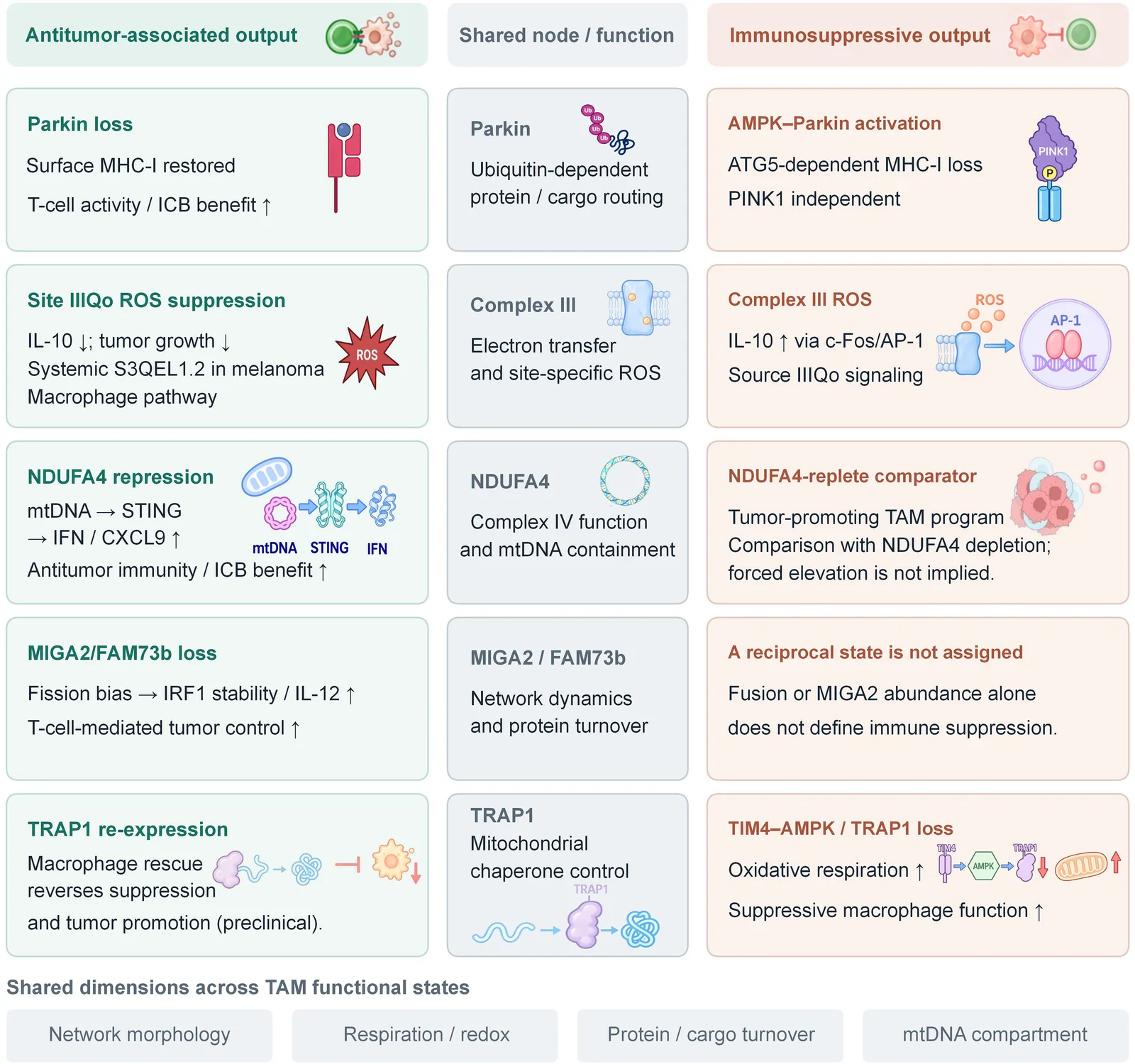
Shared mitochondrial machinery and divergent macrophage immune outputs. Rows align the same molecular node and its underlying function with the immune effects demonstrated under defined perturbations. Green and orange cells summarize antitumor-associated and immunosuppressive outputs, respectively; these columns do not represent mutually exclusive TAM populations. Parkin loss is compared with AMPK-Parkin-dependent MHC-I degradation (26), source-selective complex III ROS suppression with ROS-dependent IL-10 signaling (28, 29), and TRAP1 re-expression with TRAP1 loss (30). Systemic S3QEL1.2 treatment provides preclinical tumor evidence but does not establish macrophage-selective drug action (28). The NDUFA4 row compares repression with the NDUFA4-replete comparator described in the study (27); it does not infer that forced NDUFA4 elevation alone is sufficient to generate a suppressive state. The gray MIGA2 cell marks a limit on extrapolation: the demonstrated benefit of MIGA2/FAM73b loss and fission-associated IRF1/IL-12 signaling (25) does not make mitochondrial fusion a universal marker of immune suppression. Shared structural and biochemical dimensions are listed below the matrix. ICB, immune checkpoint blockade; IFN, interferon; ROS, reactive oxygen species. Upward and downward arrows indicate increases and decreases, respectively.

Spatial studies provide a functional context for these mechanisms. SLC2A1-positive TAM enrichment has been associated with reduced CD8 T-cell activity and resistance to PD-(L)1 blockade, with causal support from experimental perturbation (44). Chemokine-defined resident macrophage niches can instead support lymphocyte recruitment (15). Measuring macrophage location together with molecular and functional features may therefore be more informative than whole-tumor abundance alone. Direct links between most mitochondrial pathways and these spatial patterns remain to be established.

## 7 Therapeutic targeting of mitochondrial and lipid pathways

### 7.1 Mitochondrial targets supported by preclinical evidence

MQC includes potential therapeutic targets, although the same machinery is required by T cells, NK cells, dendritic cells, cardiomyocytes, neurons, hepatocytes, and tumor cells. Target selection should be linked to a macrophage function demonstrated in a relevant experimental model. MIGA2/DRP1-associated dynamics may be relevant when an IL-12-low, IRF1-low macrophage state dominates (25); Parkin inhibition may be rational when macrophages are Parkin-high and MHC-I-low but retain sufficient mitochondrial fitness (26); NDUFA4-directed remodeling may be useful when an interferon-low state can be converted through controlled mtDNA-STING signaling (27); and source-selective suppression of complex III ROS may reduce an IL-10-high program without globally inhibiting respiration (28). Earlier TAM-directed studies using CSF1/CSF1R blockade, CD40 agonism, or receptor-targeted macrophage reprogramming provide precedents for macrophage-directed cancer therapy (199–203).

TRAP1 expands the target space to mitochondrial chaperone control. Because TRAP1 loss in TAMs increased oxidative respiration and suppressive function, restoring the relevant chaperone program or interrupting its upstream TIM4-AMPK regulation could theoretically reprogram macrophages (30). However, chaperone manipulation is likely to have broad effects across cell types. Translational development will require macrophage-selective delivery and pharmacodynamic readouts showing that the intended respiratory and immune state changes occur in TAMs rather than tumor cells or lymphocytes.

### 7.2 Targeting lipid uptake and storage

The lipid axis offers upstream intervention points that may be more selective than direct ETC inhibition. CD36 blockade can reduce tumor-to-macrophage lipid transfer and metastatic support (37). DGAT1/2 inhibition can reduce LD formation and CCL20-dependent Treg recruitment in LD-laden macrophage niches (38). Targeting tumor-derived oxLDL production or TREM2 signaling may disrupt metabolically coupled cancer-cell/TAM niches (39). Candidate strategies can also target lipolysis, acyl-CoA activation, contact-site machinery, or lipid peroxidation. Each approach requires a different biomarker because reducing LD abundance, reducing lipid uptake, and reducing mitochondrial lipid flux are not equivalent interventions.

The principal risk is metabolic rerouting. Blocking LD storage without reducing lipid influx can increase lipotoxicity and ferroptotic stress. Blocking mitochondrial oxidation can divert fatty acids into other lipid species or impair macrophage survival without restoring antigen presentation. Conversely, inducing peroxidation can damage neighboring lymphocytes. Lipid-directed therapies should therefore measure isotope-traced flux, LD burden, mitochondrial respiration, lipid peroxides, efferocytosis, and the immune endpoint that motivated the intervention.

### 7.3 Cell-selective delivery and combination treatment

Macrophage-selective delivery can exploit phagocytosis, mannose or scavenger receptors, TREM2/MARCO-associated uptake, or preferential retention of particles in tumors. TLR7/8 agonist-loaded nanoparticles provide proof of principle that particles can reprogram TAMs and improve immunotherapy (204). RNA-based approaches may be particularly suited to NDUFA4 or other mitochondrial regulatory targets because they can alter expression without requiring a systemically bioavailable small molecule. Engineered mitochondrial transfer has also been proposed as a reprogramming strategy (131), although manufacturing, biodistribution, mitochondrial quality, and long-term safety remain unresolved.

Combination treatment should be informed by the macrophage function affected. Preclinical Parkin and NDUFA4 studies support combining the relevant perturbations with checkpoint blockade in the models tested (26, 27). Increased antigen presentation, IL-12 production, or CXCL9-dependent recruitment provide plausible reasons for such combinations, but do not establish efficacy across cancers. Effects on tumor cells and lymphocytes, as well as on TAMs, need to be measured.

### 7.4 Biomarkers of target engagement

A practical biomarker panel should combine macrophage identity, spatial location, mitochondrial state, and immune output. Candidate features include surface MHC-I/MHC-II, Parkin activity, NDUFA4, cytosolic mtDNA, phospho-STING, CXCL9, IL-12, IL-10, TRAP1, SLC2A1, CD36/TREM2, LD burden, and mitophagic flux. Tissue imaging is likely essential because whole-tumor RNA or circulating markers can conceal opposing niches. Spatial proximity between SLC2A1-positive TAMs and CD8 T cells, for example, can be more informative than cell abundance alone (44). Circulating mtDNA may provide complementary information but cannot distinguish whether mtDNA is producing STING-dependent antitumor signaling or TLR9-dependent suppressive signaling within tissue.

Candidate pharmacodynamic measurements include tissue imaging to identify the targeted macrophage population, organelle and metabolic assays to establish pathway engagement, and functional assays of antigen presentation, cytokine production, or lymphocyte recruitment (**Figure 4**). mt-Keima and mito-QC are research tools for genetically accessible models, rather than validated routine patient-selection assays. The proposed biomarker panel and workflow require clinical validation.

**Figure 4:**
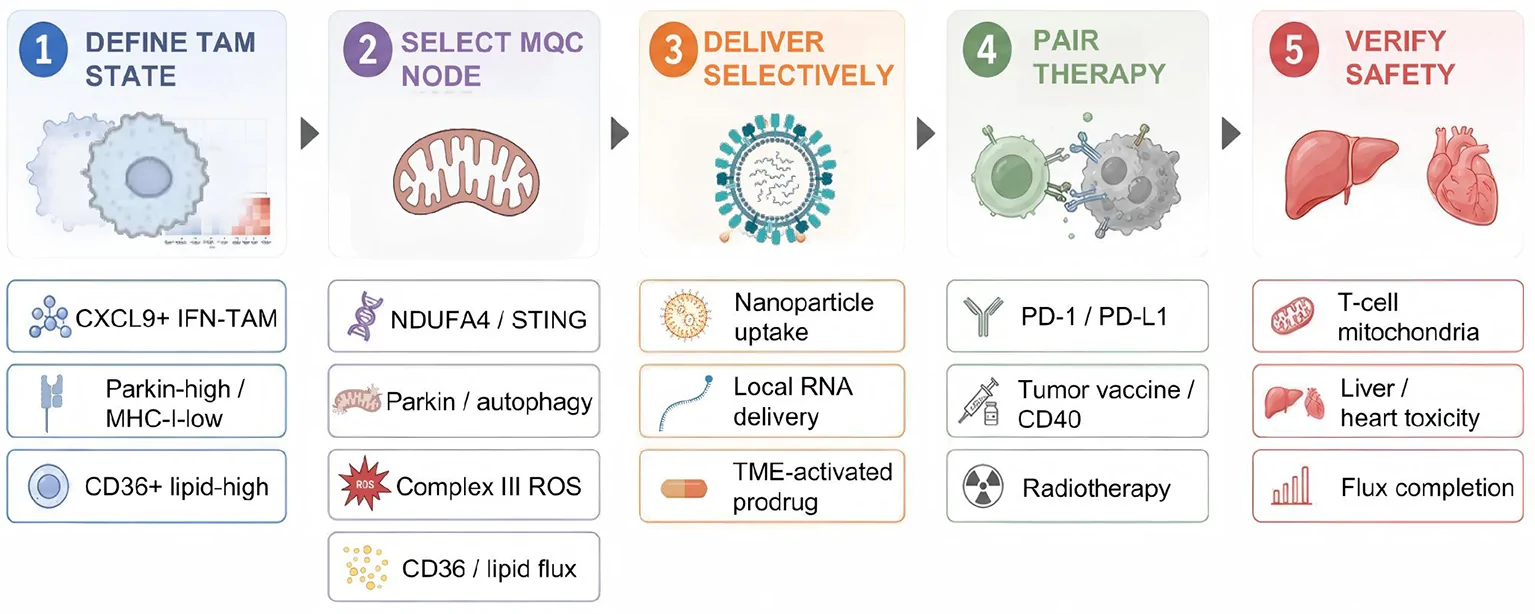
Proposed workflow for evaluating therapies that affect mitochondrial pathways in TAMs. The workflow links identification of a macrophage population and candidate target to selective delivery, treatment combinations, and pharmacodynamic assessment. Measurements include spatial macrophage identity, mitochondrial turnover, lipid metabolism, respiratory and redox state, antigen presentation, cytokines, and lymphocyte recruitment. This is a research framework; the biomarker panel and treatment-selection steps are not clinically validated. Genetically encoded flux reporters apply to experimental models rather than routine patient testing.

### 7.5 Safety and adaptive responses

Safety is a central limitation because core mitochondrial pathways are shared across tissues. Systemic DRP1, OPA1, autophagy, or ETC manipulation can impair lymphocytes and normal organs. Macrophage-selective nanoparticles, locally delivered RNA, bispecific targeting ligands, or prodrugs activated in acidic or oxidative tumor regions may provide a therapeutic window. Even with selective delivery, temporal control matters: a short period of Parkin inhibition may restore antigen presentation, whereas chronic inhibition may accumulate damaged mitochondria; transient mtDNA-STING signaling may recruit lymphocytes, whereas prolonged danger signaling can produce inflammation or selection pressure.

Resistance can also arise through metabolic substitution. TAMs may switch from glucose to fatty acids, from fatty acids to glutamine, or from mitochondrial oxidation to alternative redox systems. Spatial redistribution can mimic molecular reprogramming if one macrophage population is depleted and another enters the niche. Longitudinal sampling and lineage tracing are therefore important when a treatment changes TAM abundance. Longitudinal studies should distinguish functional changes within the targeted population from its depletion and replacement.

### 7.6 Clinical studies in related pathways

The interventions summarized above remain predominantly preclinical, and clinical studies must be classified according to what they actually test (**Table 4**). IACS-010759 directly inhibits mitochondrial complex I, but its phase I studies in relapsed/refractory acute myeloid leukemia and advanced solid tumors (NCT02882321 and NCT03291938) were systemic oncology trials. Dose-limiting lactate elevation and neurotoxicity constrained exposure, no recommended phase II dose was established, and antitumor activity was limited (205). These findings define a therapeutic-window problem rather than evidence that TAM mitochondrial inhibition is clinically effective. In the nonrandomized ATOM study (NCT02628080), atovaquone reduced tumor hypoxic volume and hypoxia-related gene expression in patients with non-small-cell lung cancer (206). This demonstrated modulation of tumor physiology, but did not establish TAM-specific mitochondrial engagement or improved survival.

**Table 4:** Preclinical interventions and clinical studies in related pathways.

Intervention	Evidence stage and model/population	Observed finding or study status	Scope of inference	Source
miR-147-based RNA therapeutics targeting Ndufa4	Preclinical; B16 melanoma	Ndufa4 repression linked complex IV remodeling and cytosolic mtDNA–STING signaling to IFN-responsive TAM programs; RNA treatment enhanced ICB activity.	Direct tumor/myeloid mechanistic evidence; no target-specific clinical efficacy established.	Clark et al. (27)
S3QEL 1.2 (site IIIQo electron-leak suppression)	Preclinical; B16F10 melanoma; systemic treatment with or without intratumoral CpG DNA	Reduced IL-10 in activated macrophages; reduced tumor growth and improved survival in mice.	Pharmacologic tumor effect was not restricted to TAMs; source-selective ROS modulation differs from global ETC inhibition.	Zotta et al. (28)
T863 + PF-06424439 (DGAT1 + DGAT2 inhibitors)	Preclinical; subcutaneous and orthotopic Hepa1–6 HCC	Combined inhibition reduced lipid-laden macrophages and Treg infiltration and delayed tumor growth.	Direct lipid/TAM evidence; systemic drug effects and mitochondrial lipid-flux dependence require separate attribution.	Wang et al. (38)
IACS-010759 (complex I inhibitor)	Phase I; relapsed/refractory AML and advanced solid tumors; NCT02882321/NCT03291938	Published trials found limited antitumor activity and dose-limiting lactate elevation/neurotoxicity; no recommended phase II dose was established.	Systemic mitochondrial oncology trials; do not establish TAM-selective mitochondrial reprogramming.	Yap et al. (205)
Atovaquone (mitochondrial complex III inhibitor)	Early phase I preoperative NSCLC study (ATOM); NCT02628080	In 30 PET-evaluable patients (15 per cohort), treated tumors showed reduced hypoxic volume and hypoxia-related transcription.	Nonrandomized tumor-oxygenation study; no survival benefit or TAM mitochondrial target engagement established.	Skwarski et al. (206)
MIW815/ADU-S100 (STING agonist)	Phase I, intratumoral monotherapy report in advanced solid tumors/lymphomas; NCT02675439	Among 47 patients, single-agent activity was limited; one confirmed partial response and evidence of systemic immune activation were reported.	Downstream DNA-sensing pathway trial; neither mitochondrial targeting nor TAM-restricted activity was demonstrated.	Meric-Bernstam et al. (207)
IMSA101 + PULSAR radiotherapy + nivolumab	Phase II SPARK; metastatic kidney cancer; NCT06601296; started April 2025	Recruiting in the registry record last posted 12 November 2025; no results posted in the record inspected 14 September 2026.	Recent pathway-level combination trial; efficacy and TAM mitochondrial target engagement remain unreported.	ClinicalTrials.gov (208)

Evidence categories refer to the experiments reported in the cited source, not to approval or predicted efficacy. Clinical mitochondrial inhibition, downstream STING agonism, and selective TAM reprogramming are different levels of evidence. Registry information was checked on 14 September 2026; SPARK was last updated on 12 November 2025. “No results posted” refers to the inspected ClinicalTrials.gov record and does not imply proven inefficacy. Reported clinical populations are the published evaluable/treated populations where specified, not registry enrollment counts.

Clinical evaluation also extends to downstream DNA-sensing pathways. Intratumoral MIW815/ADU-S100, a STING agonist, produced systemic immune-activation signals but limited single-agent activity in a phase I study (NCT02675439) (207). A more recently initiated study, SPARK (NCT06601296; actual start April 2025), is evaluating intratumoral IMSA101 with personalized ultra-fractionated stereotactic adaptive radiotherapy and nivolumab in metastatic kidney cancer (208). The inspected registry lists this phase II study as recruiting, with no results posted. Its rationale is relevant to the mtDNA–STING axis, but it does not directly test mitochondrial remodeling in TAMs. These examples should therefore be distinguished from target-specific preclinical approaches such as Ndufa4-directed RNA, S3QEL 1.2, and combined DGAT1/2 inhibition (27, 28, 38). Future translation requires paired tumor measurements that link delivery and target engagement in defined TAM subsets to mitochondrial state, immune function, and clinical outcome; pathway activation alone cannot establish this chain.

## 8 Discussion and future directions

Mitochondrial pathways regulate several TAM functions beyond energy production. Tumor studies link MIGA2-dependent dynamics to IL-12 (25), Parkin-dependent trafficking to MHC-I loss (26), NDUFA4 repression to mtDNA-STING signaling (27), complex III ROS to IL-10 (28), and TRAP1 to suppressive oxidative metabolism (30). Lipid uptake and storage also influence macrophage-associated immune suppression (37–39). Together, these findings indicate that mitochondrial morphology, mitophagy markers, or oxygen consumption alone cannot predict the immune consequence of an intervention.

An immediate priority is to connect transcriptional and spatial TAM maps (7, 15, 16) with mitochondrial function. RNA expression does not directly measure membrane potential, respiratory-complex activity, ROS production, mitophagic flux, organelle contacts, or substrate oxidation. Selected functional measurements combined with lineage and spatial information could test whether proposed states, such as Parkin-high/MHC-I-low or mtDNA-stress-high/CXCL9-high macrophages, are reproducible across tumor types and patients.

Causal studies must also resolve timing. Tumor signals may alter macrophage transcription before mitochondrial function changes, or mitochondrial perturbation may precede immune reprogramming. Inducible genetics, lineage tracing, and longitudinal measurements can help distinguish these possibilities and separate changes within a TAM population from its depletion and replacement. Comparisons across defined tumor regions are needed before assuming that a pathway has the same effect in every niche.

Another priority is to distinguish mitochondrial turnover from other functions of MQC-associated proteins. Parkin provides a direct example of this distinction. Substrate mapping and compartment-specific trafficking assays could determine whether other proteins influence immune-cargo handling independently of organelle maintenance. Mechanistic rescue experiments would help establish whether changes in antigen presentation result from mitochondrial dysfunction or from a separate trafficking pathway.

Mitochondrial genotype adds a further unresolved question. Human tumor studies now use somatic mtDNA variants to trace macrophage ancestry (137), but this does not establish that the variants cause the associated immune states. Pairing heteroplasmy-resolved lineage information with macrophage-restricted functional perturbation could separate clonal history from mitochondrial genetic effects.

Direct studies of LD-mitochondria contacts in TAMs are also needed. Multi-organelle interactions regulate fatty-acid flux and inflammatory mediator production in non-tumor macrophages (40), but their contribution in tumors remains uncertain. Imaging combined with isotope tracing and selective perturbation of contact-site proteins could test whether these interactions alter lipid oxidation, peroxidation, mitochondrial turnover, or cytokine production in TAMs.

Translation will require matching a candidate intervention to a demonstrated TAM defect. Parkin inhibition, NDUFA4 modulation, complex III ROS modulation, and lipid-directed approaches address different mechanisms and remain subject to further validation. Studies should establish effects on the intended macrophage population, possible effects on other cells, and an immune endpoint such as antigen presentation, cytokine production, lymphocyte recruitment, or ICB response. None of these molecular profiles currently defines a validated clinical selection rule.

Several limitations affect interpretation. Some mechanisms derive from infection or inflammatory models, and many tumor studies use transplantable mouse models with short treatment periods. Human evidence is often associative, while direct functional measurements in freshly isolated TAMs remain uncommon. Myeloid Cre systems can affect cells other than macrophages, metabolic inhibitors may have off-target effects, and tissue processing can alter metabolism. Lineage-resolved perturbation, rapid ex vivo or *in situ* measurements, and functional validation in human material are needed to address these limitations.

## 9 Conclusions

Mitochondrial maintenance, signaling, and lipid handling influence TAM survival and immune function. Their effects vary with the pathway, cellular context, and treatment exposure; current evidence does not establish a single optimal level of MQC. The most informative studies connect a defined mitochondrial perturbation to antigen presentation, cytokine production, or lymphocyte responses. Therapeutic development will require validation in human TAMs, selective delivery, and assessment of effects on other immune and normal cells. These requirements provide a basis for evaluating mitochondrial interventions without assuming that changes in morphology or respiration alone indicate therapeutic benefit.
